# Akt Phosphorylates HK-II at Thr-473 and Increases Mitochondrial HK-II Association to Protect Cardiomyocytes*

**DOI:** 10.1074/jbc.M113.482026

**Published:** 2013-07-08

**Authors:** David J. Roberts, Valerie P. Tan-Sah, Jeffery M. Smith, Shigeki Miyamoto

**Affiliations:** From the Department of Pharmacology, University of California San Diego, La Jolla, California 92093

**Keywords:** Akt PKB, Cell Death, Hexokinase, Insulin-like Growth Factor (IGF), Mitochondria, Phosphorylation, Cardiomyocytes, Glucose 6-Phosphate

## Abstract

Hexokinase II (HK-II) is an enzyme that catalyzes the first step in glycolysis and localizes not only in the cytosol but also at mitochondria. Akt, activated by insulin-like growth factor 1 (IGF-1) treatment in neonatal rat ventricular myocytes, translocates to mitochondria and increases mitochondrial HK-II binding. Expression of an HK-II-dissociating peptide diminished IGF-1-induced increases in mitochondrial HK-II as well as protection against hydrogen peroxide treatment, suggesting an important role of mitochondrial HK-II in IGF-1/Akt-mediated protection. We hypothesized, on the basis of an Akt phosphorylation consensus sequence present in HK-II, that Thr-473 is the target of Akt kinase activity. Indeed, recombinant kinase-active Akt robustly phosphorylates WT HK-II, but not Thr-473 mutants. Phosphomimetic (T473D)HK-II, but not non-phosphorylatable (T473A)HK-II, constitutively increased mitochondrial binding compared with WT HK-II and concomitantly confers greater protection against hydrogen peroxide. Glucose 6-phosphate (G-6P), a product of the catalytic activity of HK-II, is well known to dissociate HK-II from mitochondria. Addition of G-6P to isolated mitochondria dose-dependently dissociates WT HK-II, and this response is inhibited significantly in mitochondria isolated from cardiomyocytes expressing T473D HK-II. Pretreatment with IGF-1 also inhibits G-6P-induced overexpressed or endogenous HK-II dissociation, and this response was blocked by Akt inhibition. These results show that Akt phosphorylation of HK-II at Thr-473 is responsible for the Akt-mediated increase in HK-II binding to mitochondria. This increase is, at least in part, due to the decreased sensitivity to G-6P-induced dissociation. Thus, phosphorylation-mediated regulation of mitochondrial HK-II would be a critical component of the protective effect of Akt.

## Teaser

**Backgound:** Hexokinase II binds to mitochondria and promotes cell survival.

**Results:** Akt phosphorylates HK-II but not the threonine 473 mutant. The phosphomimetic T473D mutant decreases its dissociation from mitochondria induced by G-6P and increases cell viability against stress.

**Conclusion:** Akt phosphorylates HK-II at Thr-473, resulting in increased mitochondrial HK-II and cell protection.

**Significance:** The Akt-HK-II signaling nexus is important in cell survival.

## Introduction

Mitochondria are critical for energy generation and are also recognized as gatekeepers in the control of cell survival. Loss of mitochondrial integrity leads to necrotic or apoptotic cell death (1–4). Cardiac muscle is especially abundant in mitochondria to support the high energy demand, and mitochondrial cell death pathways play a central role in heart disease. Mitochondrial death pathways are elicited by the mitochondrial permeability transition pore (mPTP)^2^, a megachannel formed at mitochondria and/or apoptotic Bcl-2 family proteins (4–8).

Hexokinase (HK) catalyzes phosphorylation of glucose, producing glucose 6-phosphate (G-6P). Hexokinase II (HK-II) is a predominant isoform in insulin-sensitive tissues such as heart, skeletal muscle, and adipose tissues and is known to be up-regulated in many types of tumors, whereas hexokinase I (HK-I) is the main isoform in the brain but is also expressed ubiquitously (9, 10). Early studies in the 1960s demonstrated that HK-I and HK-II localize not only in cytosol, but also bind to mitochondria and that the product of HK activity, G-6P, dissociates HK from mitochondria (11, 12). Further studies revealed that mitochondrial HK binding is mediated through the N-terminal (13, 14).

There is increasing evidence that mitochondrial HKs protect mitochondria against insults (15–25). For example, it has been shown that mitochondrial HK-II antagonizes an apoptotic Bcl-2 family protein, Bax, to confer protection against apoptotic cell death (9, 20, 26) and that overexpression of HK-II in lung epithelial-like cells increases mitochondrial HK-II and prevents oxidative stress-induced cell death(15). Up-regulation of HK-II and increased mitochondrial HK-II have been suggested to contribute to cell survival in tumor cells (18, 27–30). In the heart, the binding/activity of HK-II at mitochondria is increased in response to protective interventions, including insulin, leukemia inhibitory factor, and preconditioning (19, 31, 32). In contrast, a decrease in mitochondrial HK-II is observed under stress conditions such as ischemia (33). Indeed, previous studies have demonstrated that a large dissociation of mitochondrial HK-II induces cell death (23, 34, 35). Heterozygotic HK-II knockout hearts showed no basal phenotype but are more susceptible to ischemia/reperfusion injury, which is suggested to be attributable to decreased mitochondrial HK-II (24). It has not, however, been fully understood how HK-II binding to mitochondria is regulated, especially with regard to the molecular signaling events responsible for the increase in mitochondrial HK.

Akt is an established protective kinase in many tissues. Expression of constitutively active Akt confers strong protection of the heart against ischemia/reperfusion injury (36, 37), and Akt is involved in cardiac protection induced by many receptor agonists (38). Mitochondrial HK-II has been suggested recently to be downstream of Akt. Constitutively active Akt expression in fibroblasts or receptor agonists activating Akt lead to an increase in mitochondrial HK-II activity or binding (19, 31, 32, 34, 39, 40). It has been shown that interventions dissociating HK-II from mitochondria reduce cellular protection obtained using constitutively active Akt expression (34, 39). In our previous study in cardiomyocytes, we demonstrated that activated Akt translocates to mitochondria, increasing HK-II binding to mitochondria, and that this contributes to Akt-mediated prevention of mitochondrial depolarization induced by oxidative stress. We also found that HK-II contains an Akt consensus sequence (R*XX*RS/T) conserved in mouse, rat, and human, implying that HK-II is phosphorylated by Akt (19). However, the actual phosphorylation site in HK-II for Akt has not been identified, and direct evidence that Akt-mediated phosphorylation of HK-II causes increased mitochondrial HK-II binding has not been provided.

Here we demonstrated that Thr-473 in HK-II is the phosphorylation site for Akt and that phosphorylation of HK-II at Thr-473 leads to increased association of HK-II to mitochondria. Our studies also revealed that phosphorylation of HK-II at Thr-473 decreases its dissociation from mitochondria induced by G-6P and suggests that this decrease in the product inhibition contributes to Akt-mediated increases in mitochondrial HK-II and protective effects.

## EXPERIMENTAL PROCEDURES

### 

#### 

##### Cell Culture

Neonatal rat ventricular myocytes (NRVMs) were isolated from 1- to 2-day-old Sprague-Dawley rat pups using a kit (Worthington). Myocytes were plated at a density of 3.5 × 10^4^/cm^2^ and maintained overnight in DMEM (high-glucose, Invitrogen) supplemented with 15% fetal bovine serum and antibiotics (100 units/ml penicillin and 100 μg/ml streptomycin). Cells were serum-starved for 24 h prior to the experiment. After serum starvation, cells were infected with adenoviruses and cultured for 24 h.

##### Mutagenesis and Cloning

**FIGURE 1.**
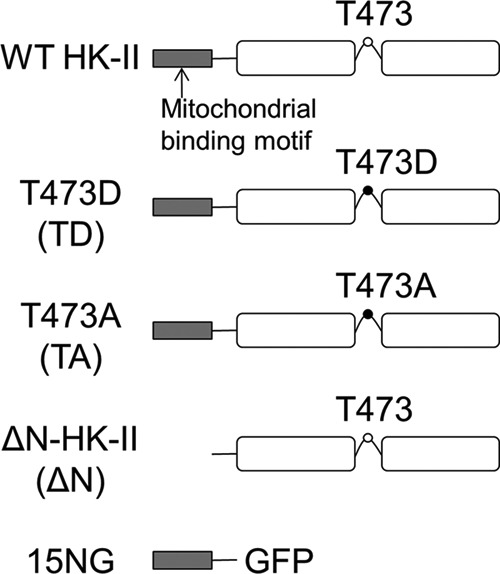
**Schematic presentation of the HK-II constructs used in this study.** *TD*, T473D; *TA*, T473A; Δ*N*, N terminus deletion mutant.

Mouse HK-II cDNA (accession no. BC054472) was obtained (Open Biosystems), amplified by PCR, and inserted into pENTR/D-TOPO (Life Sciences) for further manipulation. The Thr-473 single-point mutations and N-terminal deletion were carried out using the QuikChange site-directed mutagenesis method (Agilent Technologies). To produce 15NG, the N-terminal bases comprising the first 15 amino acids (MIASHMIACLFTELN) of HK-II were fused onto the N-terminal of GFP in pENTR. A linker sequence (GGSGG) separated the two molecules. The resulting constructs were inserted, using Gateway cloning, into the pAd/CMV/V5 DEST plasmid (Life Sciences) and were transfected into HEK293A cells for adenovirus production. These constructs are illustrated in Fig. 1.

##### Mitochondria/Cytosol Fractionation

Cardiomyocytes were washed twice with ice-cold PBS, collected in ice-cold PBS, spun down at 900 × *g* for 2 min at 4 °C, resuspended in 100 μl of hypotonic buffer (20 mm HEPES (pH 7.4)), and shaken at 600 rpm for 2 min at 4 °C. CHAPS was added to 0.1% final concentration and shaken for 5 min at 4 °C. 110 μl of buffer C (420 mm mannitol, 140 mm sucrose, 2 mm EGTA, and 20 mm HEPES (pH 7.4)) was added and spun down at 700 × *g* for 10 min to remove cell debris and nuclei. The supernatant was transferred and spun at 12,000 × *g* for 15 min at 4 °C. The supernatant was again spun down at 20,000 × *g* for 3 min, and the resultant supernatant was saved as the cytosolic fraction. The pellet was washed in buffer D (210 mm mannitol, 70 mm sucrose, 2 mm EGTA, and 20 mm HEPES (pH 7.4)) and saved as the mitochondrial fraction. For Western blotting, the isolated mitochondria were lysed in radioimmune precipitation assay buffer composed of 150 mm NaCl, 50 mm Tris (pH 7.4), 1% Nonidet P-40, 1% sodium deoxycholate, 0.1% SDS, 0.2 mm EDTA and supplemented with 200 μm Na_3_VO_4_, 10 μg/ml aprotinin, 10 μg/ml leupeptin, 1 mm PNPP, and 1 mm PMSF.

##### HK-II Release from Isolated Mitochondria

The mitochondrial pellet was resuspended in a buffer containing 120 mm KCl, 20 mm MOPS, 10 mm Tris·HCl (pH 7.4) and 0.2 mm EDTA. G-6P was added to mitochondria at various concentrations and incubated for 15 min at room temperature. The mitochondrial suspension was spun down at 20,000 × *g* for 3 min, the supernatant was transferred, and the pellet was resuspended in the buffer.

##### Western Blotting

Cardiomyocytes were washed three times with ice-cold PBS and harvested in radioimmune precipitation assay buffer. Samples were nutated at 4 °C for 10 min, spun down at 20,000 × *g* for 7 min, and then the supernatants were saved as whole cell lysates. Protein concentration was measured by Micro BCA protein assay kit (Thermo Scientific). Samples were then mixed with LDS sample buffer and reducing agent (Invitrogen), heated at 75 °C for 10 min, and then equal amounts of protein (10–30 μg) were loaded onto SDS-PAGE (Invitrogen NuPage system). Proteins were transferred to PVDF membranes (Millipore). Membranes were blocked with 5% milk in TBS-Tween for 1 h at room temperature and probed with primary antibody at 4 °C overnight at 1:1000 dilution in 5% BSA/TBS-Tween. All Western blotting antibodies used in this study were from Cell Signaling Technology. Blots were washed with TBS-Tween (5 min, 5 times) and incubated with secondary antibodies (1:2000∼1:5000 dilution) in 5% milk/TBS-Tween for 1 h.

##### Immunoprecipitation

HK-II was immunoprecipitated from whole cell lysate as described previously (19). Briefly, lysates were precleared with protein G-Sepharose for 30 min at 4 °C, and 500 μg of total protein was then incubated with 4 μg of HK-II antibody (Santa Cruz Biotechnology, C-14 antibody) in the presence of 30 μl of 50% slurry protein A/G PLUS-agarose beads (Santa Cruz Biotechnology) at 4 °C overnight. Immunocomplexes were washed with ice-cold radioimmune precipitation assay buffer three times, spun down, resuspended in 2× LDS sample buffer (Life Technology), and boiled for 5 min. Western blot analyses used PAS antibody or phosphorylated threonine antibody (Cell Signaling Technology). For the *in vitro* phosphorylation experiment, immunocomplexes were washed with ice-cold radioimmune precipitation assay buffer and resuspended in kinase reaction buffer containing 20 mm Hepes, 2 mm DTT, and 5 mm MgCl_2_ with or without 200 μm ATP and recombinant Akt (Millipore). Incubations were carried out at 30 °C for 20 min, spun down, resuspended in 2× LDS sample buffer, boiled for 5 min, and subjected to Western blotting.

##### Fluorescence Measurement in NRVMs

NRVMs were grown on glass-bottom 3.5-cm dishes and infected with adenoviruses. 24 h later, cells were loaded with 50 nm TMRE (Life Technology) for 30 min. Cells were washed twice with DPBS containing 1 mm CaCl_2_ and 1 mm MgCl_2_. Fluorescence was visualized using an Olympus LV1000 confocal microscope.

##### Cell Viability Assay

Cell viability was measured using Calcein Blue AM. Briefly, cells were loaded with 4 μm Calcein Blue AM (eBioscience) for 30 min at room temperature, washed twice with DPBS containing 1 mm CaCl_2_ and 1 mm MgCl_2_, and fluorescence was detected using an Infinite M200PRO microplate reader (TECAN).

##### Statistical Analysis

Results are reported as mean ± S.E. Comparisons of two groups were accomplished using unpaired Student's *t* test. Experiments with more than two groups were compared by analysis of variance followed by the Tukey post-hoc test. *p* < 0.05 was considered statistically significant.

## RESULTS

### 

#### 

##### Akt Activation Induced by IGF-1 Increases Association of Akt and HK-II

**FIGURE 2.**
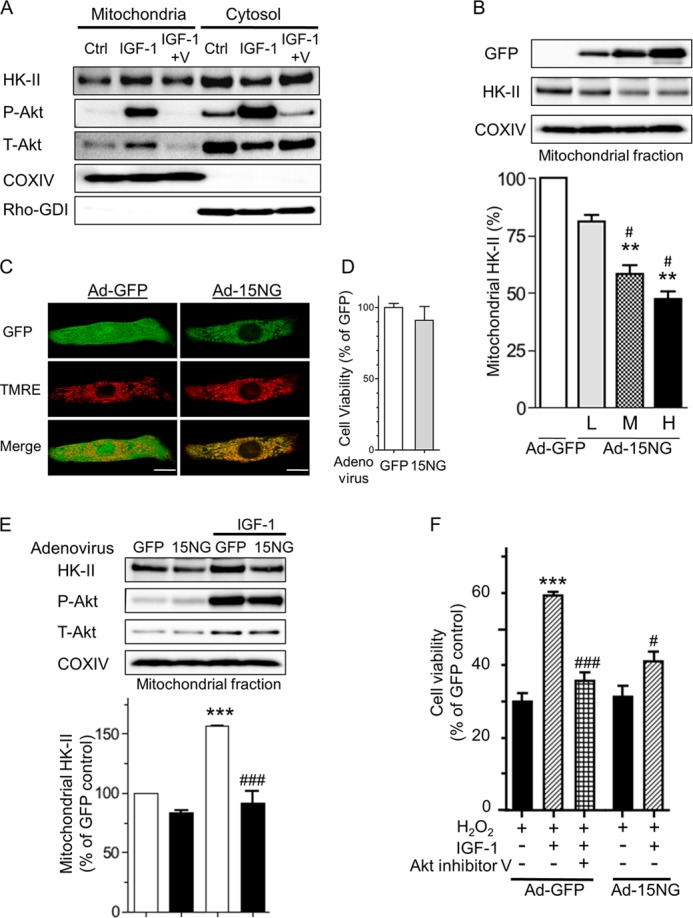
**HK-II binding to mitochondria is increased by Akt activation induced by IGF-1 and contributes to cellular protection in cardiomyocytes.** *A*, neonatal rat ventricular myocytes were treated with IGF-1 (10 nm) plus or minus Akt inhibitor V (*V*) (10 μm), fractionated into mitochondrial and cytosolic fractions, and blotted for HK-II, phosphorylated Akt (*P-Akt*), total Akt (*T-Akt*), COXIV, and Rho-GDI. COXIV and Rho-GDI antibodies were used as mitochondrial and cytosolic markers. *B*, cardiomyocytes were infected with adenovirus GFP (*Ad-GFP*) or encoding HK-II N terminus (15 amino acids) tagged with a GFP (*15NG*) construct at various MOI (low (*L*), 50; middle (*M*), 150; high (*H*), 300) for 24 h. Mitochondrial fractions were isolated and blotted for GFP, HK-II, and COXIV. **, *p* < 0.01 *versus* Ad-GFP; #, *p* < 0.05 *versus* Ad-GFP-L (50 MOI). *n* = 5–6. *C*, cells were infected with GFP adenovirus or 15NG adenovirus (*Ad-15NG*) at 50 MOI for 24 h and loaded with TMRE (50 nm) for 30 min. *Green*, GFP; *red*, TMRE. *Scale bar* = 10 μm. *D*, cells were infected with GFP adenovirus or 15NG adenovirus at 50 MOI for 24 h, and cell viability was measured by calcein-blue AM assay. *E*, cells were infected with Ad-GFP or Ad-15NG at 50 MOI for 24 h, stimulated with IGF-1 (10 nm) for 30 min, and then the mitochondrial fractions were isolated and blotted with HK-II, P-Akt, T-Akt, and COXIV. ***, *p* < 0.001 *versus* GFP control; ###, *p* < 0.001 *versus* GFP+IGF-1. *n* = 6. *F*, cells expressing GFP or 15NG were treated with 100 μm hydrogen peroxide (H_2_O_2_) for 4 h. IGF-1 (10 nm) was added 30 min before H_2_O_2_. Akt inhibitor V (tricirbine) (10 μm) was added 30 min before IGF-1. Cell viability was measured by calcein-blue AM assay. ***, *p* < 0.001 *versus* Ad-GFP + H_2_O_2_; # and ###, *p* < 0.05, 0.01 *versus* Ad-GFP + H_2_O_2_ + IGF-1. *n* = 8.

NRVMs were treated with 10 nm IGF-1 for 30 min, and the mitochondrial and cytosolic fractions were isolated and subjected to Western blotting (Fig. 2*A*). In response to IGF-1 treatment, phosphorylated and total Akt were increased in the mitochondrial fraction, which was blocked by pretreatment with Akt inhibitor V (triciribine). Mitochondrial HK-II was also increased by IGF-1, and this response was abolished by Akt inhibition. These results support our previous observations using leukemia inhibitory factor that Akt translocates to mitochondria upon activation and leads to increased mitochondrial HK-II binding in cardiomyocytes.

##### Characterization of 15NG

The HK-II N terminus peptide attached to a cell-permeable sequence (antennapedia or TAT) has been used as a method of dissociating HK-II from mitochondria to determine the role of mitochondrial HK-II (19, 20, 23, 34). In this study, we generated the HK-II N terminus (15 amino acids) tagged with GFP (15NG) and subcloned it into an adenovirus vector. Cells were infected with Ad-15NG at different MOI (50, 150, and 300 MOI), and the expression of the peptide was assessed by Western blotting for GFP after 24 h of infection. The expression of the peptide was MOI-dependently increased in the mitochondrial fraction, and mitochondrial HK-II was decreased concomitantly, confirming the ability of the N terminus peptide to dissociate HK-II from mitochondria (Fig. 2*B*). Using cells infected at a low MOI, we characterized the intracellular distribution of 15NG *versus* untagged GFP together with TMRE to detect active mitochondria. As shown in Fig. 2*C*, GFP fluorescence in cells infected with Ad-15NG was colocalized with TMRE fluorescence (indicative of an intact mitochondrial membrane potential). However, cells infected with Ad-GFP showed diffuse fluorescence throughout the cell, demonstrating preferential localization of 15NG to mitochondria. Cell viability was not affected by the expression of 15NG at this level of expression (Fig. 2*D*). Thus, at a low level of expression, the dissociating peptide is unlikely to affect mitochondrial integrity and, thus, cell survival.

##### Inhibiting IGF-1-induced HK-II Mitochondrial Association Inhibits Protection

To determine whether the expression of 15NG inhibits the IGF-1-induced increase in HK-II binding to mitochondria, cells were infected with GFP (control) or 15NG adenovirus at 50 MOI, stimulated with IGF-1, and then the mitochondrial fractions were isolated (Fig. 2*E*). IGF-1 treatment increased total Akt, phospho-Akt, and HK-II in the mitochondrial fraction. 15NG expression diminished the increase in HK-II in the mitochondrial fraction but did not affect total and phospho-Akt levels. To determine whether the increase in HK-II binding to mitochondria contributes to IGF-1-mediated cell protection, cardiomyocytes were treated with 100 μm hydrogen peroxide for 4 h, and cell viability was measured (Fig. 2*F*). IGF-1 treatment increased cell viability against hydrogen peroxide in cardiomyocytes, and this protection was blocked by inhibition of Akt, suggesting a critical role of Akt in IGF-1-mediated protection. This IGF-1/Akt-induced protection was greatly attenuated in cells expressing 15NG. These results support the notion that increased mitochondrial HK-II contributes to Akt-mediated protection against oxidative stress in cardiomyocytes.

##### Akt Phosphorylates HK-II at Thr-473

**FIGURE 3.**
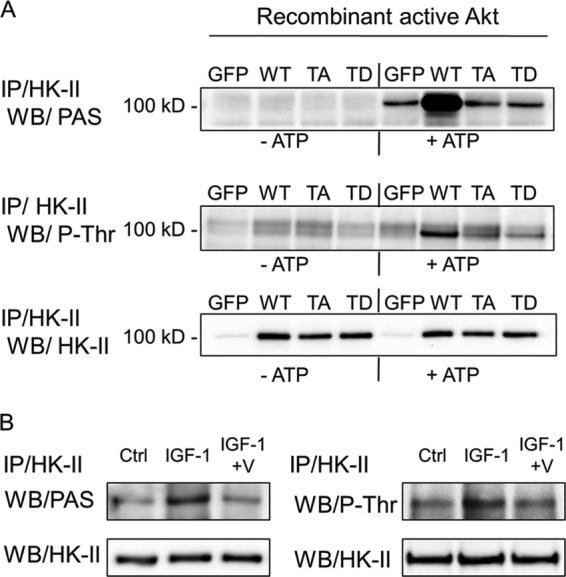
**Akt phosphorylates HK-II at Thr-473.** *A*, HK-II was immunoprecipitated (*IP*) from cells expressing GFP, WT HK-II, T473A (*TA*) HK-II, or T473D (*TD*) HK-II, resuspended in kinase reaction buffer, and then recombinant kinase active Akt (ΔPH, S473D Akt) was added in the presence or absence of ATP. After 20 min of incubation at 30 °C, samples were centrifuged and resuspended in 2× LDS buffer and subjected to Western blotting for PAS, P-Thr, or HK-II. *B*, endogenous HK-II was immunoprecipitated from NRVMs, stimulated with IGF-1 (10 nm) ± Akt inhibitor V (*V*) (10 μm, 30-min pretreatment), and subjected to Western blotting for PAS, P-Thr, or HK-II. *Ctrl*, control.

To test the hypothesis that Thr-473 in the Akt consensus sequence found in HK-II is the phosphorylation site for Akt, we generated the phosphomimetic T473D (*TD*) and non-phosphorylatable T473A mutants (*TA*) (Fig. 3*A*). GFP (control), WT, T473D, and T473A were expressed adenovirally in cells, and HK-II was immunoprecipitated. The immunoprecipitates were then subjected to *in vitro* phosphorylation using recombinant kinase-active Akt (ΔPH, S473D) in the presence or absence of ATP. Phosphorylation of HK-II was assessed by PAS antibody (phosphorylated Akt consensus sequence antibody) or P-Thr antibody. Recombinant active Akt catalyzed a robust ATP-dependent phosphorylation of WT HK-II but did not increase phosphorylation of Thr-473 mutants over control (endogenous HK-II) levels, providing direct evidence that Thr-473 in HK-II is phosphorylated by Akt. To determine whether Akt, activated by IGF-1, phosphorylates endogenous HK-II, cells were treated with IGF-1 with or without Akt inhibitor V. HK-II was then immunoprecipitated and blotted using PAS or P-Thr antibody (Fig. 3*B*). IGF-1 treatment increased PAS or P-Thr signals, which were blocked by Akt inhibitor V, confirming that HK-II is phosphorylated by Akt.

##### Phosphorylation of HK-II at Thr-473 Increases Mitochondrial HK-II Binding

**FIGURE 4.**
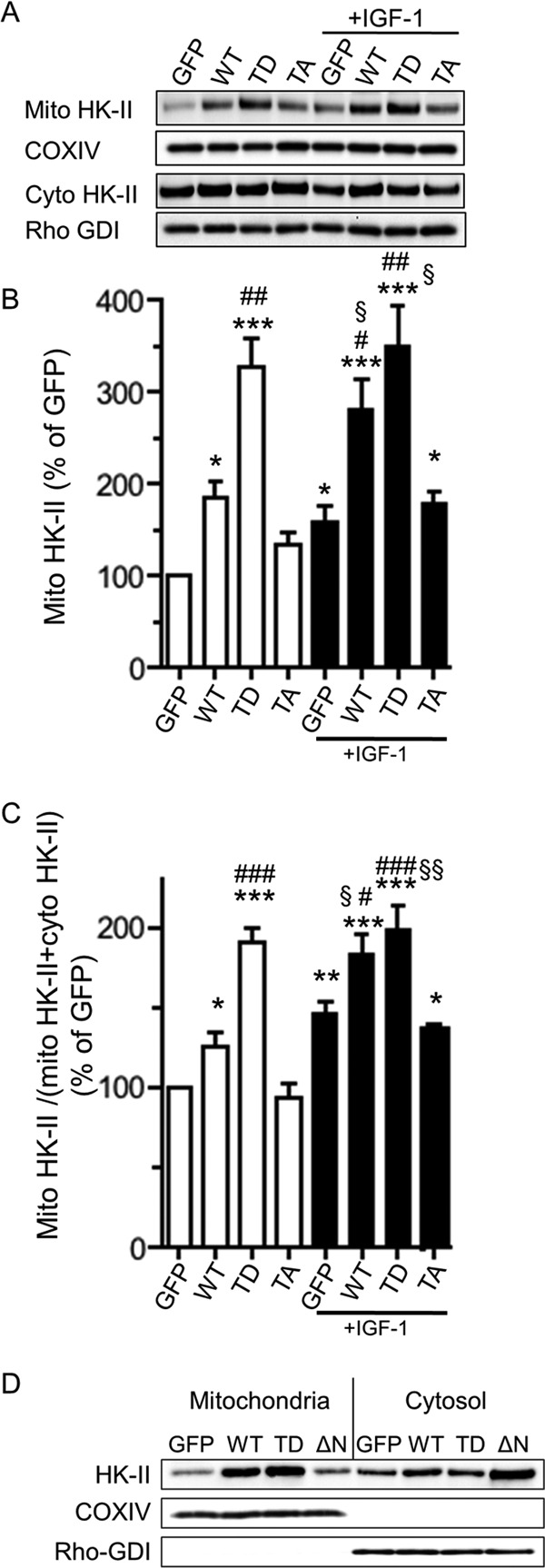
**Phosphomimetic mutant (T473D) HK-II but not non-phosphorylatable mutant (T473A) increases mitochondrial association.** Cells were infected with GFP, WT, T473D (*TD*), or T473A (*TA*) adenovirus, and mitochondrial and cytosolic fractions were isolated. *A*, representative Western blot analyses of mitochondrial (*Mito*) and cytosolic (*Cyto*) HK-II. Some cells were also stimulated with 10 nm IGF-1 for 30 min prior to the fractionation. *B*, quantitative analysis of the amount of HK-II in the mitochondrial fractions. * and ***, *p* < 0.05, 0.001 *versus* GFP control; # and ##, *p* < 0.05, 0.01 *versus* WT; §, *p* < 0.05 *versus* GFP + IGF-1. *n* = 8–9. *C*, quantitative analysis of the ratio of mitochondrial HK-II to total (mitochondria + cytosol) HK-II. *, ** and ***, *p* < 0.05, 0.01, and 0.001 *versus* GFP control; # and ###, *p* < 0.05 and 0.001 *versus* WT; §, *p* < 0.05 *versus* GFP + IGF-1. *n* = 8–9. *D*, mitochondria were isolated from cells expressing GFP, WT HK-II, T473D, HK-II, or N terminus deletion mutant (Δ*N*) HK-II and subjected to Western blotting for HK-II, VDAC, and Rho-GDI. VDAC and Rho-GDI were used as mitochondrial and cytosolic markers respectively.

We then evaluated the mitochondrial binding of WT, T473D, and T473A HK-II (Fig. 4*A*). Overexpression of WT, T473D, or T473A increased the amount of HK-II in the mitochondrial fraction. Slight increases were observed in the cytosolic fractions. T473D showed a significantly larger increase in the mitochondrial fraction compared with WT or T473A (Fig. 4, *A* and *B*). The effect of IGF-1 treatment was examined. Similar to Fig. 2*D*, IGF-1 increased endogenous or overexpressed WT HK-II in the mitochondrial fraction, and IGF-1 treatment did not change mitochondrial HK-II in T473D HK-II expressing cells. Nor did it increase the mitochondrial HK-II in T473A-expressing cells over control (GFP) cells, corresponding to endogenous HK-II phosphorylation and association.

Although the MOI of each adenovirus was chosen carefully to achieve equivalent protein expression, higher expression can result in more mitochondrial HK-II. To normalize the difference in the protein expression levels of each construct, the ratio of mitochondrial HK-II to total HK-II (mitochondrial + cytosolic HK-II) is quantified in Fig. 4*C*. The WT showed a slight but significant increase in HK-II mitochondrial binding over the control. Remarkably, the T473D mutant showed a robust increase in mitochondrial HK-II, whereas the T473A mutant did not increase the ratio over the control. These results suggest that phosphorylation of Thr-473 sensitizes HK-II to associate with mitochondria. IGF-1 treatment increased the ratio in GFP- and WT-expressing cells. In cells expressing T473D, the ratio was not increased by IGF-1 treatment. The ratio in T473A-expressing cells was increased by IGF-1, but this response was similar to control cells treated with IGF-1.

The N terminus of HK-II has been considered to mediate its binding to mitochondria. The N terminus deletion mutation (ΔN HK-II), GFP, WT, or T473D were overexpressed in NRVMs, and the cells were fractionated (Fig. 4*D*). Western blotting demonstrated that mitochondrial HK-II was not increased in cells expressing ΔN HK-II despite high overexpression and that IGF-1 was ineffective in promoting mitochondrial binding of ΔN HK-II (data not shown), confirming that the N terminus is essential and sufficient for HK-II binding to mitochondria.

##### Increased Mitochondrial HK-II Binding Confers Cardiomyocyte Protection against Hydrogen Peroxide

**FIGURE 5.**
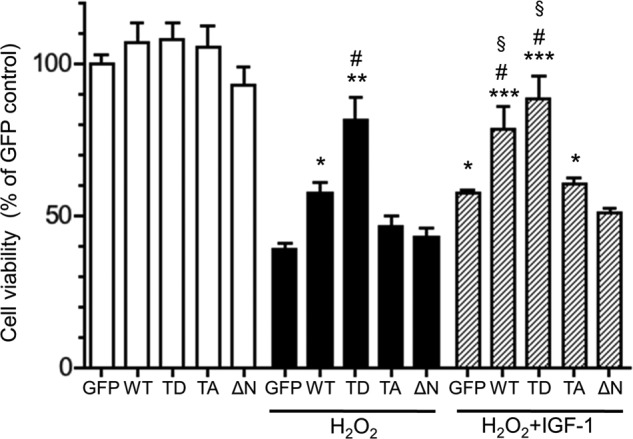
**Phosphomimetic mutant (T473D) HK-II but not non-phosphorylatable mutant (T473A) enhances cellular protection against hydrogen peroxide.** Cells expressing GFP, WT HK-II, T473D (*TD*) HK-II, T473A (*TA*) HK-II, or N terminus deletion mutant (Δ*N*) HK-II were treated with 100 μm hydrogen peroxide (H_2_O_2_) for 4 h, and cell viability was determined by calcein-blue AM assay. Some cells were also pretreated with 10 nm IGF-1 for 30 min prior to hydrogen peroxide treatment. *, **, ***, *p* < 0.05, 0.01, 0.001 *versus* GFP + H_2_O_2_; #, *p* < 0.05 *versus* WT + H_2_O_2_; §, *p* < 0.05 *versus* GFP + IGF-1 + H_2_O_2_. *n* = 8.

To determine whether phosphorylation-dependent increases in mitochondrial HK-II binding support cell survival in cardiomyocytes, cells were infected with WT, T473D, T473A, and ΔN HK-II, treated with hydrogen peroxide, and then cell viability was assessed (Fig. 5). WT HK-II overexpression resulted in an increase in cell viability compared with the control (GFP). In parallel with the mitochondrial binding, T473D HK-II greatly enhanced the protection over WT, whereas enhanced cell protection was not observed in T473A- or ΔN HK-II-expressing cells. IGF-1 treatment increased cell viability in GFP- and WT HK-II-expressing cells. Correlating with mitochondrial HK-II binding, T473D-mediated protection was not increased further by IGF-1, and the effect of IGF-1 on T473A was comparable with that of the IGF-1-treated control. IGF-1 also failed to significantly increase cell viability in cells expressing ΔN HK-II. Together, these results show that phosphorylation of HK-II increases mitochondrial binding, which provides the protective effect against oxidative stress in cardiomyocytes.

##### Phosphorylation of HK-II at Thr-473 Decreases G-6P-dependent Inhibition of HK-II Mitochondrial Binding

We next investigated the mechanism by which phosphorylation at Thr-473 by Akt may increase the amount of HK-II at mitochondria.

**FIGURE 6.**
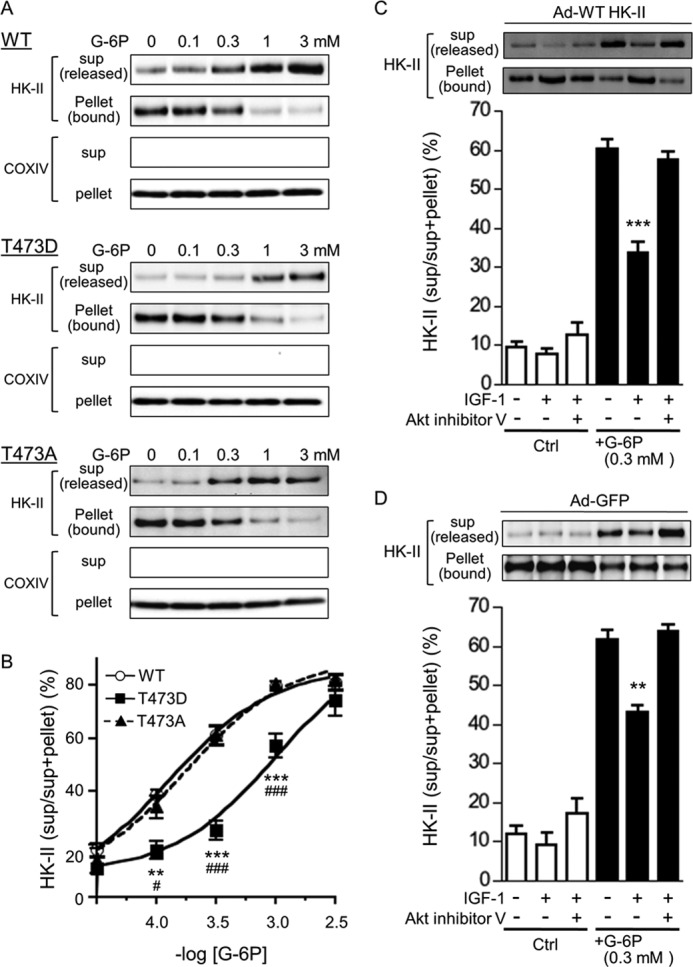
**The phosphomimetic mutant (T473D) or phosphorylation of HK-II mediated by Akt inhibits G-6P-induced mitochondrial HK-II dissociation.** *A*, mitochondria were isolated from cells expressing WT HK-II, T473D, or T473A HK-II; treated with G-6P; and incubated for 15 min. Mitochondria were spun down, and HK-II in the supernatant (*sup*) (dissociated HK-II) and that in the pellet (mitochondria-bound HK-II) were blotted. COXIV was used as loading control. *B*, dose-response curves of G-6P-dependent HK-II dissociation from mitochondria. The ratio of supernatant HK-II to total (supernatant + pellet) HK-II was plotted against the logarithm of the dose of G-6P. ** and ***, *p* < 0.01 and 0.001 T473D *versus* WT; # and ###, *p* < 0.05 and 0.001 T473D *versus* T473A. *n* = 5. *C*, Akt activation elicited by IGF-1 inhibits WT HK-II dissociation from mitochondria induced by G-6P. Cells were infected with WT HK-II adenovirus. Some cells were treated with 10 nm IGF-1 for 30 min in the presence or absence of 10 μm Akt inhibitor V. Mitochondria were isolated, treated with 0.3 mm G-6P for 15 min, centrifuged, and then the supernatant and the pellet were subjected to Western blotting. ***, *p* < 0.001 *versus* WT + G-6P. *n* = 5. *D*, Akt activation elicited by IGF-1 inhibits endogenous HK-II dissociation from mitochondria induced by G-6P. Cells were treated with 10 nm IGF-1 for 30 min in the presence or absence of 10 μm Akt inhibitor V, and then mitochondria were isolated and treated with 0.3 mm G-6P for 15 min. **, *p* < 0.01 *versus* WT + G-6P. *n* = 4.

It has been established that G-6P can dissociate HK-II from mitochondria, and we hypothesized that phosphorylation of HK-II counteracts the dissociating effect of G-6P. To test the hypothesis, mitochondria were isolated from cells expressing WT or T473D HK-II, incubated with G-6P for 15 min, and the supernatant (dissociated HK-II) and pellet (mitochondria-bound HK-II) were subjected to Western blotting (Fig. 6*A*). As expected, G-6P dose-dependently dissociated WT HK-II from mitochondria up to ∼80% at 3 mm. Remarkably, this response was decreased significantly, and the dose-dependent curve was shifted rightward, in mitochondria isolated from cells expressing T473D HK-II (Fig. 6*B*). On the other hand, the T473A mutant did not inhibit G-6P-dependent dissociation of HK-II from mitochondria (Fig. 6, *A* and *B*). The level of COXIV in the pellet was unchanged, nor was it detected in the supernatant, suggesting that HK-II release was not due to disruption of mitochondria. Similar dose-dependent curves were obtained in mitochondria supplemented with succinate and rotenone (data not shown).

To further determine the effect of Akt-mediated phosphorylation of HK-II on G-6P-dependent dissociation, cells overexpressing WT HK-II were treated with IGF-1 in the presence or absence of an Akt inhibitor, and mitochondria were isolated and treated with 0.3 mm G-6P for 15 min (Fig. 6*C*). G-6P-induced HK-II release to the supernatant was inhibited significantly by IGF-1 treatment and was reversed by pretreatment with an Akt inhibitor, suggesting Akt mediated inhibition of G-6P-dependent HK-II dissociation. The effect of G-6P on the dissociation of endogenous HK-II was also tested (Fig. 6*D*). After 30 min of IGF-1 treatment in the presence or absence of Akt inhibitor V, mitochondria were isolated, and the effect of 0.3 mm G-6P was examined. G-6P-induced dissociation was inhibited significantly in cells treated with IGF-1, and this inhibitory effect was abolished by inhibition of Akt.

## DISCUSSION

**FIGURE 7.**
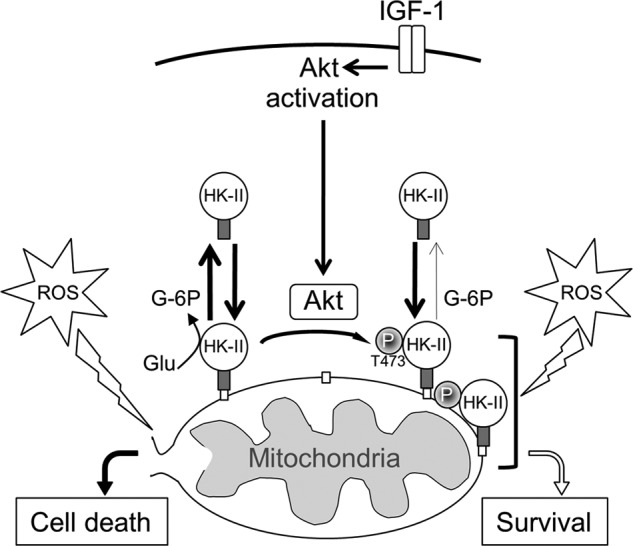
**Schematic illustrating how Akt-mediated phosphorylation of HK-II at Thr-473 inhibits G-6P-dependent dissociation, leading to increased HK-II mitochondrial binding and cellular protection against ROS.**

Although it has been demonstrated that an increased level of HK-II at mitochondria is protective and is increased by protective interventions but decreased under stress, it has not been fully determined which molecular signals regulate the level of HK-II at mitochondria. Here we demonstrate, for the first time, that Thr-473 in HK-II is phosphorylated by Akt and that this phosphorylation leads to increases in mitochondrial HK-II binding through inhibition of G-6P-dependent dissociation, conferring resistance to oxidative stress (Fig. 7).

Overexpression of WT HK-II increases mitochondrial HK-II and confers protection against hydrogen peroxide, which is enhanced significantly in HK-II T473D-expressing cells, whereas ΔN HK-II, lacking the ability to bind to mitochondria, does not confer protection. Conversely, mitochondrial HK-II dissociation peptide (15NG) inhibits the IGF-1-mediated increase in mitochondrial HK-II and cellular protection. These results strongly support the protective effect of an increase in mitochondrial HK-II against stress (15–25). Although we did not see deleterious effects of the 15NG-dissociating peptide at 50 MOI, higher expression of the peptide significantly reduces basal HK-II in the mitochondrial fraction and induces cell death (data not shown). These results support previous reports demonstrating that TAT-attached HK-II-dissociating peptide dose-dependently dissociates HK-II and that a large detachment of HK-II from mitochondria rapidly causes mitochondrial depolarization because of the opening of the mPTP and cell death in the heart (23, 35). Together, these results imply that association of HK-II with mitochondria is an important regulator of the integrity of mitochondria in cardiomyocytes.

The mechanisms by which HK-II prevent opening of the mPTP are not fully understood. Previous studies have suggested a link between HK-II, a negative regulator of the mPTP, and cyclophilin D, a positive regulator (35, 41, 42). Although several protective mechanisms for mitochondrial HK-II have been suggested, such as an antioxidant effect and Bax antagonism, pore-forming core component(s) of the mPTP remain unidentified, and, thus, further studies will be required to delineate the mechanism for HK-II mediated mitochondrial protection.

It has been demonstrated that Akt, upon its activation at the plasma membrane, translocates to different subcellular compartments, including the nucleus and mitochondria (19, 40, 43, 44). In this study, we also observed that IGF-1 activates and translocates Akt to mitochondria in cardiomyocytes, conferring protection against hydrogen peroxide-induced cell death. Additionally, a FRET reporter of Akt activity has also revealed the presence of active Akt at the mitochondria of non-cardiac cells (45, 46). Thus, Akt redistribution to mitochondria can be stimulated by various agonists and is functionally relevant in different cell types. We also showed previously that Akt activity is regulated locally at mitochondria by an Akt-directed phosphatase, PHLPP1 (40). As a result of its mitochondrial distribution, it has been reported that Akt raises mitochondrial tolerance to stress, although the target molecules of Akt at mitochondria have not been fully determined. We and others have previously suggested a functional connection between Akt activation and the increase in mitochondrial HK-II in promoting cell survival (19, 20, 26, 34, 39), although the molecular mechanism by which Akt regulates mitochondrial HK-II had not been determined.

We confirmed that Akt activation induced by IGF-1 phosphorylates and increases mitochondrial binding of HK-II (Figs. 3 and 4). Using Thr-473 mutants of HK-II, we demonstrate that Akt phosphorylates HK-II at Thr-473 in the Akt consensus sequence of HK-II, and our finding that the T473D but not the T473A mutant shows a higher association with mitochondria than WT HK-II further suggests that the phosphorylation of Thr-473 is responsible for the Akt-mediated increase in HK-II mitochondrial binding. This is further supported by our findings that IGF-1 increases WT HK-II association with mitochondria but not Thr-473 mutants. Likewise, IGF-1 enhances WT HK-II-mediated protection but does not affect cell survival in cells expressing Thr-473 mutants. The importance of mitochondrial binding of HK-II in Akt-mediated protection is supported by our observation that IGF-1 does not confer cardioprotection over control in cells expressing the ΔN mutant. Although our data suggest that phosphorylation of HK-II is sufficient for increasing mitochondrial HK-II binding, it has been reported that GSK3β, a constitutively active kinase phosphorylated and inhibited by Akt, blocks HK-II binding to mitochondria in HeLa cells (47). Thus, Akt appears to positively regulate HK-II binding to mitochondria by direct phosphorylation of HK-II and indirect mechanisms via inhibition of GSK3β (47, 48) to preserve mitochondrial integrity against cellular stress.

It has been well established that G-6P induces dissociation of HK-II from mitochondria (11, 18, 49–51). In considering the mechanism by which phosphorylation of HK-II at Thr-473 leads to an increase in mitochondrial HK-II binding, we determined whether dissociation of HK-II induced by G-6P was affected by phosphorylation. We found that mitochondrial binding of WT HK-II is inhibited in the range of 0.1–3 mm, which is in agreement with G-6P concentration increases of ∼0.17 to ∼1.3 mm in perfused working heart (52) and with the reported IC_50_ of G-6P *versus* HK-II (53, 54). Our finding that the T473D mutant is more resistant to G-6P-dependent dissociation than the WT or the non-phosphorylatable mutant (T473A, Fig 6, *A* and *B*) strongly suggests that phosphorylation of Thr-473 decreases the sensitivity of HK-II to G-6P-induced mitochondrial dissociation and, thereby, increases mitochondrial HK-II. Importantly, Akt activation stimulated by IGF-1 also inhibits exogenous WT and endogenous HK-II dissociation induced by G-6P. To our knowledge, this is the first presentation that the inhibitory effect of G-6P on mitochondrial HK-II binding is negatively regulated by posttranscriptional modification. There was no difference in G-6P-induced inhibition of the catalytic activity of HK-II in WT, T473D, and T473A.^3^ Thus, Thr-473 phosphorylation appears not to influence G-6P binding to HK-II but stabilizes the HK-II-mitochondrial interaction against G-6P.

G-6P is known to be accumulated in the heart during ischemia about 10-fold (55, 56), and a burst of ROS formation at the onset of reperfusion has been well established to induce cell death through opening of the mPTP (1, 3, 4). Counteracting the activation of this mitochondrial death pathway, reperfusion injury salvage kinases, including Akt, are activated in response to reperfusion (57). It has also been suggested that preconditioning, a series of brief cycles of ischemia/reperfusion prior to a sustained period of ischemia, confers strong cardioprotection (5), and preconditioning has been demonstrated to increase mitochondrial HK-II (32). Together, Akt-mediated phosphorylation of HK-II and the resultant increased resistance to G-6P-dependent dissociation from mitochondria would contribute to an established protective effect of Akt against ischemia/reperfusion injury in the heart.

The Pederson laboratory (18, 27, 58) has suggested that mitochondrial HKs lead to enhancement of ATP production because of preferential access of the enzyme to mitochondrially generated ATP, contributing to high glycolytic activity in tumor cells (Warburg effect) in which both Akt and HK-II are often up-regulated. Akt is also well recognized as metabolic regulator, supporting cellular growth (37, 59, 60). Interestingly, it has been reported that activated Akt translocates to mitochondria and enhances bioenergetics in non-cardiac cells and cardiomyocytes (61, 62). Thus, phosphorylation-mediated regulation of HK-II binding to mitochondria could also provide metabolic support to the cell under growth conditions. Further study will be required to determine whether phosphorylation of HK-II plays a role in the increase in energy metabolism regulated by Akt under growth conditions. It has also been reported that insulin treatment in adipose and skeletal muscle cell lines increases HK-II mRNA and protein levels through Akt pathways (63, 64). The transcriptional up-regulation of HK-II and posttranscriptional regulation of intracellular localization of HK-II might be an important component of Akt-mediated cellular growth and protection.

In conclusion, we demonstrate that Akt phosphorylates HK-II at Thr-473, inducing increases in HK-II binding to mitochondria and conferring resistance to cytotoxic insult. This is attributable, at least in part, to decreased sensitivity of phosphorylated HK-II to G-6P-induced dissociation. We suggest that this regulation of mitochondrial HK-II binding by Akt-mediated phosphorylation plays a crucial role in cell survival.

## Footnotes

3 D. J. Roberts, V. P. Tan-Sah, J. M. Smith, and S. Miyamoto, unpublished observations.

## Footnotes

2 The abbreviations used are:

mPTP: mitochondrial permeability transition pore
HK: hexokinase
G-6P: glucose 6-phosphate
NRVM: neonatal rat ventricular myocite
PAS: phosphorylated Akt consensus sequence antibody
IGF: insulin-like growth factor
MOI: multiplicity of infection
Ad: adenovirus
p-Thr: phosphorylated threonine
15NG: N-terminal HK-II with GFP
PNPP: *p*-nitrophenyl phosphate
TMRE: tetramethylrhodamine ethyl ester
DPBS: Dulbecco's phosphate-buffered saline
TAT: transactivator of transcription
COXIV: cytochrome *c* oxidase IV
Rho-GDI: Rho GDP-dissociation inhibitor
VDAC: voltage-dependent anion channel.
